# antiSMASH 3.0—a comprehensive resource for the genome mining of biosynthetic gene clusters

**DOI:** 10.1093/nar/gkv437

**Published:** 2015-05-06

**Authors:** Tilmann Weber, Kai Blin, Srikanth Duddela, Daniel Krug, Hyun Uk Kim, Robert Bruccoleri, Sang Yup Lee, Michael A Fischbach, Rolf Müller, Wolfgang Wohlleben, Rainer Breitling, Eriko Takano, Marnix H Medema

**Affiliations:** Novo Nordisk Foundation Center for Biosustainability, Technical University of Denmark, Hørsholm, Denmark; Department of Microbial Natural Products, Helmholtz Institute for Pharmaceutical Research Saarland, Helmholtz Centre for Infection Research, Saarland University, Saarbrücken, Germany; German Centre for Infection Research (DZIF), Location Hannover-Braunschweig, Germany; Department of Chemical and Biomolecular Engineering (BK21 Plus Program) / BioInformatics Research Center, Korea Advanced Institute of Science and Technology, Daejeon, Republic of Korea; Congenomics, LLC, Glastonbury, CT, USA; Department of Bioengineering and Therapeutic Sciences/California Institute for Quantitative Biosciences, University of California, San Francisco, USA; Interfaculty Institute for Microbiology and Infection Medicine, Eberhard Karls University of Tübingen, Tübingen, Germany; German Center for Infection Research (DZIF), Location Tübingen, Germany; Manchester Centre for Synthetic Biology of Fine and Speciality Chemicals (SYNBIOCHEM), Manchester Institute of Biotechnology, Faculty of Life Sciences, The University of Manchester, Manchester, UK; Microbial Genomics and Bioinformatics Research Group, Max Planck Institute for Marine Microbiology, Bremen, Germany; Bioinformatics Group, Wageningen University, Wageningen, The Netherlands

## Abstract

Microbial secondary metabolism constitutes a rich source of antibiotics, chemotherapeutics, insecticides and other high-value chemicals. Genome mining of gene clusters that encode the biosynthetic pathways for these metabolites has become a key methodology for novel compound discovery. In 2011, we introduced antiSMASH, a web server and stand-alone tool for the automatic genomic identification and analysis of biosynthetic gene clusters, available at http://antismash.secondarymetabolites.org. Here, we present version 3.0 of antiSMASH, which has undergone major improvements. A full integration of the recently published ClusterFinder algorithm now allows using this probabilistic algorithm to detect putative gene clusters of unknown types. Also, a new dereplication variant of the ClusterBlast module now identifies similarities of identified clusters to any of 1172 clusters with known end products. At the enzyme level, active sites of key biosynthetic enzymes are now pinpointed through a curated pattern-matching procedure and Enzyme Commission numbers are assigned to functionally classify all enzyme-coding genes. Additionally, chemical structure prediction has been improved by incorporating polyketide reduction states. Finally, in order for users to be able to organize and analyze multiple antiSMASH outputs in a private setting, a new XML output module allows offline editing of antiSMASH annotations within the Geneious software.

## INTRODUCTION

The secondary metabolism of bacteria and fungi is a rich source of bioactive chemical compounds with great potential for pharmaceutical, agricultural and nutritional applications. For example, in the field of antiinfectives, almost 70% of the drugs currently in medical use are such secondary metabolites or their derivatives (1). The genes encoding the biosynthetic pathways that are responsible for the production of these secondary metabolites are usually clustered together on the chromosome in biosynthetic gene clusters (BGCs). In recent years, genome mining of such BGCs has become a key methodology to identify new molecules, leading to the discovery of dozens of novel compounds. A variety of computational tools have been developed to support scientists in this field. Most of the available tools are dedicated to the analysis of specific classes of secondary metabolites. For example, ClustScan (2), NP.searcher (3) and SBSPKS (4) focus on non-ribosomal peptide and polyketide biosynthesis pathways, while BAGEL3 (5) focuses on ribosomally synthesized and post-translationally modified peptides (RiPPs). While most tools primarily focus on prokaryotic pathways, SMURF (6) also addresses fungal secondary metabolite producers. For a comprehensive review of tools for the genomic analysis of secondary metabolism, see Weber (7). Since 2011, the antibiotics and Secondary Metabolite Analysis SHell (antiSMASH) has served as a comprehensive web server and a stand-alone tool for the automatic genomic identification and analysis of BGCs of any type, thus facilitating rapid genome mining of a wide range of bacterial and fungal strains (8,9). Here, we report the third version of antiSMASH, which has undergone several key improvements.

## NEW FEATURES AND UPDATES

### Integration with ClusterFinder

A key limitation to the original antiSMASH BGC detection algorithm was that, despite many major classes of secondary metabolites being covered by its detection logic (see Supplementary Tables S1 and S2), it was still limited to the detection of known types of BGCs. To overcome this limitation, the ClusterFinder algorithm was recently published (10). ClusterFinder uses a hidden Markov model to probabilistically predict BGC-like regions in genomes based on the frequencies of observed PFAM domains inside and outside a comprehensive set of known BGCs. The key assumption of the algorithm is that even the biosynthetic pathways for unknown compound families that are very different from known secondary metabolites utilize the same broad enzyme families (e.g., oxidoreductases and methyltransferases) for the catalysis of key reactions. While ClusterFinder has a somewhat higher false positive rate than the original version of antiSMASH, it has been shown to effectively identify BGCs for entirely new classes of chemicals (10).

From a user perspective, the most convenient way to analyze genomes using ClusterFinder is to inspect its results alongside those of antiSMASH, so that all detailed classification and analysis features offered by antiSMASH are included in the workflow. Hence, we fully integrated ClusterFinder into antiSMASH, including detailed input variables to tune the sensitivity and specificity. Also, the clusters detected by ClusterFinder are further categorized into saccharide (‘Cf_saccharide’), fatty acid (‘Cf_fatty_acid’) and putative (‘Cf_putative’) biosynthetic types, according to the classification rules defined by Cimermancic *et al*. (10). Altogether, the antiSMASH web server now provides access to both a very specific algorithm that can accurately detect BGCs belonging to a large number of known classes and a highly sensitive algorithm that effectively identifies potentially novel types of BGCs.

### Dereplication and comparison with known pathways

**Figure 1.**
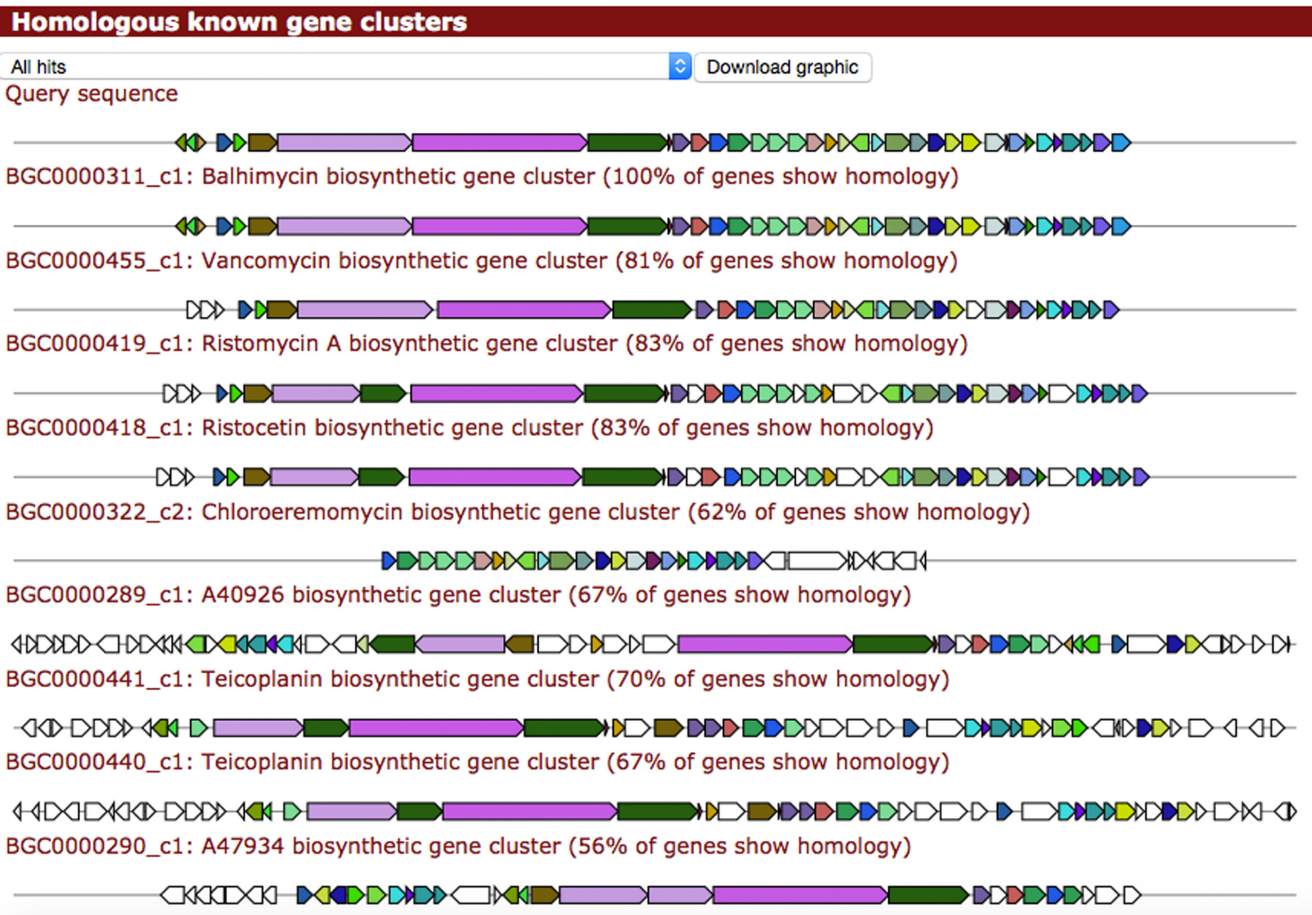
Example output of a KnownClusterBlast output, using the balhimycin gene cluster (GenBank Y16952.3). The significance thresholds used are the same as for the ClusterBlast module (8). Following the balhimycin gene cluster itself, several other BGCs involved in the biosynthesis of similar glycopeptides are shown as next best hits. The percentage of genes in the query cluster that are present in the hit cluster is included as extra information. Also, hyperlinks to the MIBiG repository are available, where users can find additional information on each gene cluster.

Another key new feature in antiSMASH is a dedicated module to compare identified BGCs with those encoding the biosynthetic pathways for known compounds. Previously, this was partially possible using the built-in ClusterBlast module, but as the output contains hits against all BGCs in the database, it is often not immediately obvious whether a BGC has been experimentally characterized or not. Hence, we have now included a dedicated KnownClusterBlast module (Figure 1) that compares identified BGCs with the comprehensive dataset of known BGCs (currently 1172 in total) from the ‘Minimum Information about a Biosynthetic Gene cluster’ (MIBiG) community project (Medema *et al*., submitted for publication; see http://mibig.secondarymetabolites.org for a full overview and additional info on these BGCs). This is a very important feature as (i) dereplication of existing compounds is crucial for effective discovery of novel natural products instead of finding the same molecules repeatedly, and (ii) comparative analysis of unknown and known gene clusters may provide hints concerning the function of certain genes within the cluster, inferred from homology.

### Identification and analysis of enzyme active sites

With the new ‘Active Site Finder’ module, it is possible to identify and annotate conserved amino acid motifs, for example active sites or product-determining key residues of biosynthetic enzymes. The active sites are annotated in the exported Genbank and/or EMBL files. Identified active sites are also displayed in the ‘Gene’ drop-down windows or in the cluster details view of the antiSMASH results web page. The motifs are defined in an XML file that can easily be extended on local antiSMASH installations. Currently, the following motifs are recognized by the active site finder module: active sites for ketosynthase (KS) domains, acyltransferase (AT) domains, dehydratase (DH) domains, ketoreductase (KR) domains, acyl-carrier protein (ACP) domains, thioesterases and cytochrome P450 oxygenases; and predictions based on key residues: ACP-type beta-branching/non-branching, ketoreductase domain (D-/L-) stereochemistry and enoyl reductase domain (2S/2R) stereochemistry.

### Improvements in chemical structure prediction

Another important feature of antiSMASH has been its ability to display chemical structures of secondary metabolites predicted from the annotated BGCs. In this new version, we made two major improvements to generate more precise chemical structures by considering the effects of ketoreductase, dehydratase and enoylreductase that influence the redox status of keto groups in polyketides, and trans-AT type type I PKS logic. These corrections laid a foundation for further improvement in the display of more sophisticated structures of secondary metabolites. As before, predicted structures for NRPS and PKS clusters assume NRPS/PKS co-linearity and do not yet predict possible cyclizations or other post-NRPS/-PKS tailoring reactions.

### Future developments toward genome-scale metabolic modeling of secondary metabolite biosynthesis

In the next phase of antiSMASH development, one major focus will be the automated integration of the predicted secondary metabolite biosynthesis pathways into genome-scale models of metabolism (11,12). As a first step in this direction, antiSMASH 3.0 already includes an interface to EFICAz2.5, which predicts Enzyme Commission (EC) numbers for all the metabolic genes in the submitted genome (13). In addition, a prototype functionality has been added for the automated generation of prokaryotic draft genome-scale metabolic models, following the established approach of homology-based modeling (14). This will be the basis for further developments toward the integration of secondary metabolic pathways (Kim H.U. *et al*., unpublished results).

### BiosynML output for offline editing

**Figure 2.**
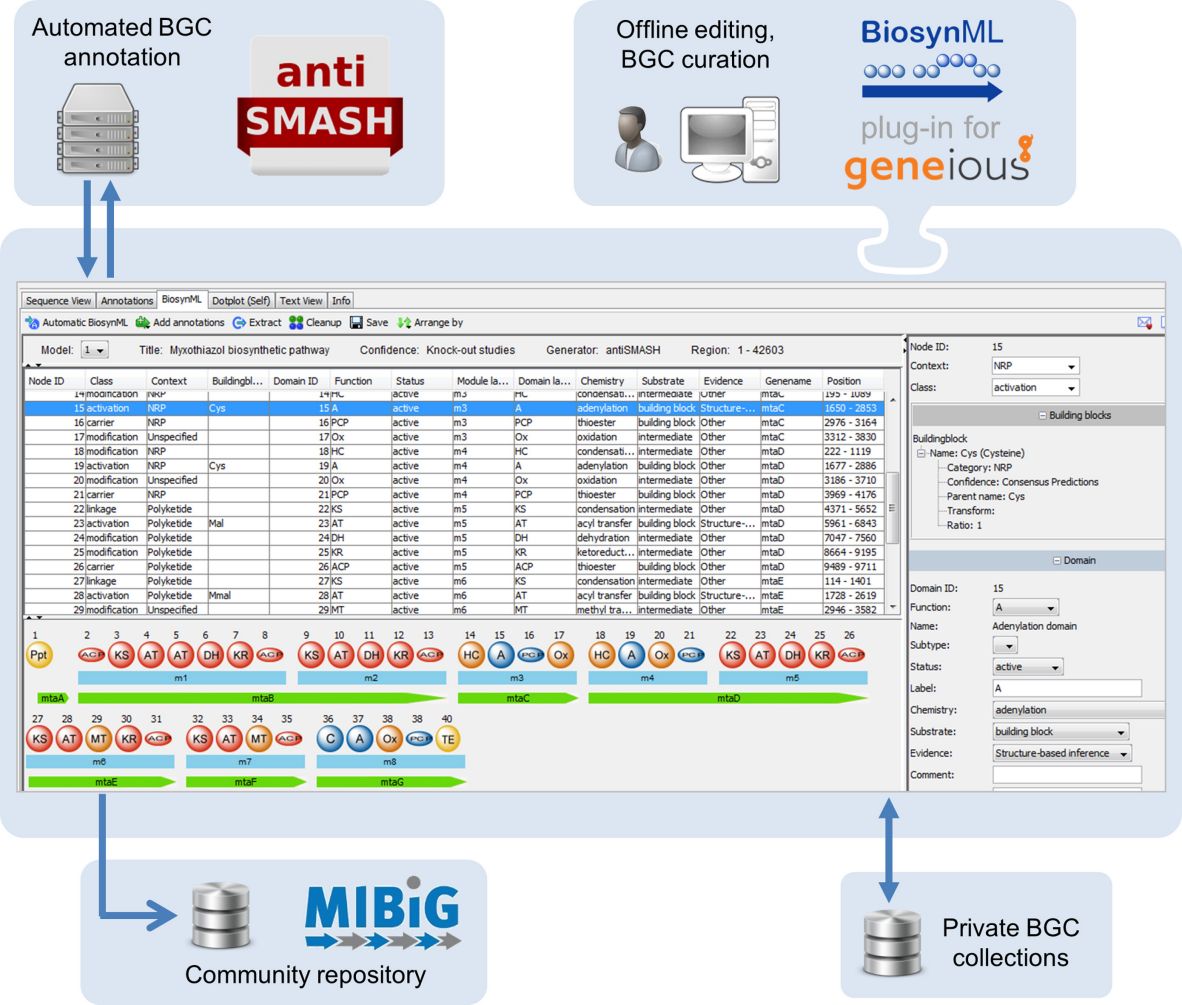
BiosynML output and Geneious plugin. The schematic shows the interfacing of typical tasks during BGC analysis—including antiSMASH annotation, manual BGC refinement, deposition to in-house databases and submission to the public MIBiG repository—supported by BiosynML functionality.

Researchers usually submit single gene clusters, complete genomes or collections of scaffolds to antiSMASH. Hence, the antiSMASH results are frequently not endpoints of their workflow, but are used as input for additional downstream analyses and may also undergo manual curation. We have added the BiosynML output module to export detailed antiSMASH analysis results in XML format, which serves as a container for interfacing to custom analysis workflows and also enables offline archiving of results. The use of BiosynML output is exemplified by a plugin (available from http://www.biosynml.de/) for the widely used desktop software Geneious, thereby allowing users to import antiSMASH-annotated sequences, to organize their pathway collection and to manually refine gene cluster information (Figure 2) (15). Typical tasks performed during offline editing of pathways include the manual assignment of incorporated building blocks on the basis of experimental evidence, grouping of biosynthetic domains into functional modules (typically for modular PKS and NRPS gene clusters) and additional domain-specific analysis, e.g. by sequence alignments and inspection of signature motifs. In addition, the BiosynML plugin can handle direct antiSMASH job submission and result retrieval. Moreover, it also assists with the deposition of MIBiG-compliant gene clusters in the course of the ongoing MIBiG community initiative (Medema *et al*., submitted for publication). Finally, the BiosynML format aims to facilitate the prototyping of custom bioinformatic workflows by providing structured access to antiSMASH annotation results.

### Improved nomenclature and detection of RiPPs and polyketides

For a number of compound classes, such as polyketides and RiPPs, we have updated the nomenclature and corresponding detection logic (Supplementary Table S1). For lanthipeptides, we have improved the prediction of modification reactions, lanthionine bridge count and finished peptide masses (9). For other RiPPs, the community-agreed nomenclature recently published by van der Donk *et al*. (16) has been adopted. For polyketides and lipids, specific BGC classes have been added that are responsible for the biosynthesis of (dialkyl)resorcinols (17), aryl polyenes (10), ladderane lipids (18) and polyunsaturated fatty acids (19). Also, new domain types have been added for the domain representation of modular polyketide synthases and non-ribosomal peptide synthetases, including, e.g. branching, crotonase and pyran synthase domains (Supplementary Table S3). These updates ensure that the antiSMASH output pages reflect the latest developments in the field.

### Back-end and library updates

The job management and dispatch infrastructure (https://bitbucket.org/antismash/runsmash) of the antiSMASH web server (https://bitbucket.org/antismash/websmash) have been redesigned to be more flexible when adding new features to antiSMASH and to increase speed and scalability. While the old MySQL-based job queue has reliably served around 90,000 jobs since the antiSMASH 1.0 publication in July 2011, the number of jobs submitted per month is still steadily increasing. In the seven weeks since the start of 2015, the new Redis-based queue has handled over 6000 jobs already, while being more flexible when adding new features to antiSMASH. A number of third-party tools, libraries and databases used by antiSMASH were updated to their latest version: BioPython (20) 1.65, PFAM database (21) 27.0 and NCBI BLAST+ (22) 2.2.30. The ClusterBlast database was updated to include clusters detected from the latest GenBank release. Additionally, the ClusterBlast routines now use DIAMOND (23) to calculate the cluster comparisons more quickly.

## CONCLUSIONS AND FUTURE PERSPECTIVES

With the newly introduced features, antiSMASH is now even more comprehensive than it was before (Table 1), and will be useful for the discovery of new secondary metabolites as well as for metabolic engineering (24). Still, there are several important future challenges ahead. For example, chemical structure prediction is at the moment still limited to the ‘core’ peptides and polyketides that are off-loaded from modular assembly-lines, while cyclization and tailoring reactions are difficult to accurately predict. Perhaps a combinatorial strategy will make this possible in the near future, leading to a result consisting of multiple possible end compounds (as previously done to some extent in NP.searcher (3)). Another important remaining challenge is to scale up antiSMASH to allow the simultaneous analysis of large numbers of genomes and metagenomes; the development of automated BGC networking (10,25,26) could be a key technology to make this possible. In the coming years, we will strive to continuously upgrade antiSMASH in order to incorporate the latest insights and technologies, so that natural product researchers will always have access to a state-of-the-art tool for the comprehensive identification and analysis of BGCs. We invite the community to join us in these efforts by contributing new algorithms and analysis tools as antiSMASH plugins. This will ensure that the community as a whole will benefit from an integrated and centralized online usage environment.

**Table 1.** Overview of analyses integrated into antiSMASH

**Rule-based detection of BGCs^a,b^**	
Aminocoumarins	Melanins
Aminoglycosides/aminocyclitols	Microcins
Aryl polyenes	Microviridins
Bacteriocins	Non-ribosomal peptides
Beta-lactams	Nucleosides
Bottromycins	Oligosaccharide
Butyrolactones	Others
ClusterFinder fatty acids	Phenazines
ClusterFinder saccharides	Phosphoglycolipids
Cyanobactins	Phosphonates
(Dialkyl)resorcinols	Polyunsaturated fatty acids
Ectoines	Trans-AT type I PKS
Furans	Type I PKS
Glycocins	Type II PKS
Head-to-tail cyclized peptides	Type III PKS
Heterocyst glycolipid PKS-like	Proteusins
Homoserine lactones	Sactipeptides
Indoles	Siderophores
Ladderane lipids	Terpenes
Lantipeptides	Thiopeptides
Linear azol(in)e-containing peptides (LAPs)	
Lasso peptide	
Linaridins	
**Rule-independent detection of BGCs**	
ClusterFinder	
**Cluster-specific analyses**	
Domain structure of PKSs and NRPSs^c^	
NRPS: A-domain specificity prediction	
PKS: AT specificity prediction	
Identification of conserved active site motifs; stereochemistry-determining motifs	
Prediction of core chemical structure (NRPS, PKS, lanthipeptides)	
smCOG secondary metabolism-related gene family prediction	
**Genome-wide analyses**	
Protein family detection (PFAM) search	
EC number prediction	
Homology-based metabolic modeling (with template models *Escherichia coli* /*Streptomyces coelicolor*)	
**Genome comparisons**	
ClusterBlast (identification of similar clusters in sequenced genomes)	
SubClusterBlast (identification of conserved operons and multigene modules with known function)	
KnownClusterBlast (identification of similar experimentally characterized gene clusters)	
**Links to other Web-resources**	
NCBI BLAST+	
NaPDoS	
Norine	
**Output file formats**	
Genbank	
EMBL	
SBML (for metabolic model files)	
BiosynML	
XLS (Microsoft Excel)	
Tab-delimited text files	
**Input file formats**	

FASTA (nucleotide or protein).

Genbank/Genpept.

EMBL.

Direct download via NCBI accession number.

^a^For a list of profile Hidden Markov Models (pHMMs) used to detect the different classes, please see Supplementary Table S1.

^b^For a list of rules, please see Supplementary Table S2.

^c^For a list of detectable domains, please see Supplementary Table S3.

## AVAILABILITY

http://antismash.secondarymetabolites.org// This website is free and open to all users and there is no login requirement. Source code is available from https://bitbucket.org/antismash/antismash/.

## Supplementary Material

Supplementary Data
