# Supplementary material for: antiSMASH 3.0—a comprehensive resource for the genome mining of biosynthetic gene clusters
Source: Nucleic Acids Res. 2015 May 6;43(Web Server issue):W237–43. doi: 10.1093/nar/gkv437 (PMC4489286; doi:10.1093/nar/gkv437)
Supplement: Supplementary Data [file gkv437_supp.pdf]

Supplementary material for

antiSMASH 3.0 – a comprehensive resource for the genome mining of biosynthetic gene clusters

Tilmann Weber, Kai Blin, Srikanth Duddela, Daniel Krug, Hyun Uk Kim, Robert Bruccoleri, Sang Yup Lee, Michael A. Fischbach, Rolf Müller, Wolfgang Wohlleben, Rainer Breitling, Eriko Takano and Marnix H. Medema

Content

Table S1: Signature HMMs for detection of secondary metabolite biosynthesis gene clusters

Table S2: Rules for detection of secondary metabolite biosynthesis gene clusters

Table S3: pHMM models for domains of modular NRPS/PKS enzymes

Supplementary references

Table S1: Signature HMMs for detection of secondary metabolite biosynthesis gene clusters (extended according to Medema et al. 2011, Blin et al. 2013)

Compound class	Description	HMM name	Source
NRPS	Condensation domain	Condensation	PFAM PF00668.13
NRPS	Adenylation domain	AMP-binding	PFAM PF00501.21
NRPS	Adenylation domain with integrated oxidase	A-OX	Medema et al. (2011)
NRPS/PKS	Thiolation domain	PP-binding	PFAM PF00550.18
PKS	Ketosynthase domain	PKS_KS	SMART
PKS	Acyltransferase domain	PKS_AT	SMART
PKS	Trans-acyltransferase docking domain	ATd	Medema et al. (2011)
PKS (neg.)	Bacterial type I fatty acid synthase	bt1fas	Medema et al. (2011)
PKS (neg.)	Fungal type I fatty acid synthase	ft1fas	Medema et al. (2011)
PKS (neg.)	Type II fatty acid synthase	t2fas	Medema et al. (2011)
PKS (neg.)	FabH fatty acid synthase	fabH	Medema et al. (2011)
PKS (neg.)	FabF fatty acid synthase	FabF	This study.
PKS	Enediyine ketosynthase	ene_KS	Yadav et al. (2009)
PKS	Modular ketosynthase	mod_KS	Yadav et al. (2009)
PKS	Hybrid ketosynthase	hyb_KS	Yadav et al. (2009)
PKS	Iterative ketosynthase	itr_KS	Yadav et al. (2009)
PKS	Trans-AT ketosynthase	tra_KS	Yadav et al. (2009)
PKS	Unusual PKS HglD-like	hglD	Medema et al. (2011)
PKS	Unusual PKS HglE-like	hglE	Medema et al. (2011)
PKS	Type II PKS ketosynthase	t2ks	Medema et al. (2011)
PKS	Other stand-alone ketosynthase	t2ks2	Medema et al. (2011)
PKS	Type II PKS Chain length factor	t2clf	Medema et al. (2011)
PKS	Type III PKS N-terminal	Chal_sti_synt_N	PFAM PF00195.12
PKS	Type III PKS C-terminal	Chal_sti_synt_C	PFAM PF00195.12
PKS	Aryl polyene ketosynthase 1	APE_KS1	This study.
PKS	Aryl polyene ketosynthase 2	APE_KS2	This study.
PKS	DarB-like (dialkyl)resorcinol-associated ketosynthase	DarB	This study.
PKS	CerJ-like KSIII enzyme	ksIII	This study.
PKS	Ladderane lipid ketosynthase	ladderane	This study.

Compound class	Description	HMM name	Source
PKS	Polyunsaturated fatty acid ketosynthase	PUFA_KS	This study.
Terpene	Terpene synthase C terminal	Terpene_synth_C	PFAM PF03936.9
Terpene	Terpene sythase	Terpene synth	PFAM PF01397.14
Terpene	Phytoene_synthase	phytoene_synth	Medema et al. (2011)
Terpene	Lycopene cyclase	Lycopene_cycl	PFAM PF05834.5
Terpene	Terpene cyclase	terpene_cyclase	Medema et al. (2011)
Terpene	NapT7-like protein	NapT7	Medema et al. (2011)
Terpene	Fungal geranylgeranyl pyrophosphate synthase	fung_ggpp	Medema et al. (2011)
Terpene	Fungal geranylgeranyl pyrophosphate synthase, model 2	fung_ggpp2	Medema et al. (2011)
Terpene	Dimethylallyl tryptophan synthase	dmat	Medema et al. (2011)
Terpene	Trichodiene synthase	trichodiene_synt	Medema et al. (2011)
Lanthipeptides	LanC-like Lantibiotics biosynthesis protein	LANC_like	PFAM PF05147.6
Lanthipeptides	Lantibiotic dehydratase, N-terminus	Lant_dehyd_N	PFAM PF04737.6
Lanthipeptides	Lantibiotic dehydratase, C-terminus	Lant_dehyd_C	PFAM PF04738.6
Lanthipeptides	Lantibiotic antimicrobial peptide 18	Antimicrobial18	PFAM PF08130.4
Lanthipeptides	Gallidermin	Gallidermin	PFAM PF02052.8
Lanthipeptides	Lantibiotic, type A	L_biotic_typeA	PFAM PF04604.6
Lanthipeptides	Lantibiotic, gallidermin/nisin family	TIGR03731	TIGR03731
Lanthipeptides	Lantibiotic leader lacticin 481 group	LE-LAC481	De Jong et al. (2010)
Lanthipeptides	Lantibiotic leader mersacidin cinnamycin group	LE-MER+2PEP	De Jong et al. (2010)
Lanthipeptides	Lantibiotic leader LanBC modified	LE-LanBC	De Jong et al. (2010)
Lanthipeptides	Lantibiotic peptide lacticin 481 group	MA-LAC481	De Jong et al. (2010)
Lanthipeptides	Lantibiotic peptide nisin epidermin group	MA-NIS+EPI	De Jong et al. (2010)
Lanthipeptides	Lantibiotic peptide nisin group	MA-NIS	De Jong et al. (2010)
Lanthipeptides	Lantibiotic peptide epidermin group	MA-EPI	De Jong et al. (2010)
Lanthipeptides	Lantibiotic peptide two	MA-2PEPA	De Jong et al.

Compound class	Description	HMM name	Source
	component alpha		(2010)
Lanthipeptides	Lantibiotic peptide two component beta	MA-2PEPB	De Jong et al. (2010)
Lanthipeptides	lantibiotic peptide lacticin 481 group (dufour et al)	LE-DUF	De Jong et al. (2010)
Lanthipeptides	lantibiotic leader lacticin 481 group (dufour et al)	MA-DUF	De Jong et al. (2010)
Lanthipeptides	FxLD family lantipeptide	TIGR04363	TIGR04363
Lanthipeptides	*Streptomyces* PEQAXS motif lantipeptide	Strep_PEQAXS	Blin et al. (2013)
Lanthipeptides	Flavoprotein	flavoprotein	PFAM PF02441
Lanthipeptides	Tryptophane halogenase	Trp_halogenase	PFAM PF04830
Lanthipeptides	Phosphate kinase	Pkinase	PFAM PF00069
Lanthipeptides	Lantibiotic-associated domain DUF4135	DUF4135	PFAM PF13575
Lanthipeptides	P450 oxygenase	p450	PFAM PF00067
Lanthipeptides	Short-chain dehydrogenase	adh_short	PFAM PF00105
Lanthipeptides	Short-chain dehyrdrogenase, C-terminus	adh_short_C2	PFAM PF13561
Bacteriocin	Putative *Streptomyces* bacteriocin	strepbact	Medema et al. (2011)
Bacteriocin	Antimicrobial peptide 14	Antimicrobial14	PFAM PF08109.4
Bacteriocin	Bacteriocin_IIc domain	Bacteriocin_IIc	PFAM PF10439.2
Bacteriocin	Bacteriocin_IId domain	Bacteriocin_IId	PFAM PF09221.3
Bacteriocin	Bacteriocin_IIdc_cydomain	BacteriocIIc_cy	PFAM PF12173.1
Bacteriocin	Bacteriocin_II domain	Bacteriocin_II	PFAM PF01721.11
Bacteriocin	Bacteriocin_IIi domain	Bacteriocin_IIi	PFAM PF11758.1
Bacteriocin	Lactococcin	Lactococcin	PFAM PF04369.6
Bacteriocin	Antimicrobial peptide 17	Antimicrobial17	PFAM PF08129.4
Bacteriocin	Lactococcin 972	Lactococcin_972	PFAM PF09683.3
Bacteriocin	Lactococcin G-beta	LcnG-beta	PFAM PF11632.1
Bacteriocin	Cloacin	Cloacin	PFAM PF03515.7
Bacteriocin	Linocin M18	Linocin_M18	PFAM PF04454.5
Bacteriocin	Bacteriocin biosynthesis cyclodehydratase	TIGR03603	TIGR03603
Bacteriocin	Bacteriocin biosynthesis docking scaffold	TIGR03604	TIGR03604
Bacteriocin	SagB-type dehydrogenase	TIGR03605	TIGR03605
Bacteriocin	Bacteriocin, circularin A/uberolysin famil	TIGR03651	TIGR03651
Bacteriocin	Bacteriocin, microcyclamide/patellamide family	TIGR03678	TIGR03678
Bacteriocin	Thiazole-containing bacteriocin maturation	TIGR03693	TIGR03693

Compound class	Description	HMM name	Source
	protei		
Bacteriocin	Bacteriocin propeptide	TIGR03798	TIGR03798
Bacteriocin	Bacteriocin biosynthesis cyclodehydratase	TIGR03882	TIGR03882
Bacteriocin	Bacteriocin, BA_2677 family	TIGR03601	TIGR03601
Bacteriocin	Bacteriocin protoxin, streptolysin S family	TIGR03602	TIGR03602
Bacteriocin	Two-chain TOMM family	TIGR03795	TIGR03795
Bacteriocin	NHLP leader peptide domain	TIGR03793	TIGR03793
Bacteriocin	Bacteriocin maturation radical SAM protein 1	TIGR03975	TIGR03975
Bacteriocin	Thuricin prepeptide	thuricin	Blin et al. (2013)
Bacteriocin	Small prepeptide associated domain	DUF692	PFAM DUF692
Head-to-tail cyclized peptide	Subtilosin A	Subtilosin_A	PFAM PF11420.1
Head-to-tail cyclized peptide	SkfC biosynthesis enzyme	skfc	Blin et al. (2013)
Microviridin	Microviridin A	mvnA	Medema et al. (2011)
Microviridin	Marinostatin/microviridin prepeptide	mvd	Blin et al. (2013)
Thiopeptide	Thiostrepton-like thiopeptides	thiostrepton	Medema et al. (2011)
Sactipeptide	Putative subtilosin biosynthesis enzyme ywiA	subtilosin	Blin et al. (2013)
Linaridin	Cypermycin prepeptide	cypermycin	Blin et al. (2013)
Linaridin	Cypermycin biosynthesis cypI enzyme	cypI	Blin et al. (2013)
Lasso peptide	Lasso peptide modification enzyme	lasso	Blin et al. (2013)
Microcin	Microcin J25 prepeptide	micJ25	Blin et al. (2013)
Microcin	Microcin J25 processing protein McjC	mcjC	Blin et al. (2013)
Glycocin	Glycocin prepeptide	glycocin	Blin et al. (2013)
Glycocin	Sublancin prepeptide	sublancin	Blin et al. (2013)
Bottromycin	Bottromycin biosynthesis enzyme	botH	Blin et al. (2013)
Proteusin	Proteusin prepeptide	PoyD	This study.
Beta-lactams	Beta-lactam synthase	BLS	Medema et al. (2011)
Beta-lactams	Clavulanic acid synthase-like	CAS	Medema et al. (2011)
Beta-lactams	Tabtoxin synthase-like	Tabtoxin	Medema et al. (2011)
Aminoglycosides /	2-deoxy-scyllo-inosose	DOIS	Medema et al.

Compound class	Description	HMM name	Source
aminocyclitols	synthase		(2011)
Aminoglycosides / aminocyclitols	NeoL-like deacetylase	neoL_like	Medema et al. (2011)
Aminoglycosides / aminocyclitols	SpcD-/SpcK-like thymidylyltransferas	spcDK_like_glyc	Medema et al. (2011)
Aminoglycosides / aminocyclitols	SpcF-/SpcG-like glycosyltransferase	spcFG_like	Medema et al. (2011)
Aminoglycosides / aminocyclitols	StrH-like glycosyltransferase	strH_like	Medema et al. (2011)
Aminoglycosides / aminocyclitols	StrK-like phosphatase	strK_like1	Medema et al. (2011)
Aminoglycosides / aminocyclitols	StrK-like phosphatase	strK_like2	Medema et al. (2011)
Aminoglycosides / aminocyclitols	ValA-like 2-epi-5-epi-valiolone synthase	valA_like	Medema et al. (2011)
Aminoglycosides / aminocyclitols	2-epi-5-epi-valiolone synthase, SalQ-like	salQ	Medema et al. (2011)
Aminocoumarins	NovK-like reductase	novK	Medema et al. (2011)
Aminocoumarins	NovJ-like reductase	novJ	Medema et al. (2011)
Aminocoumarins	NovI-like cytochrome P450	novI	Medema et al. (2011)
Aminocoumarins	NovH-like protein	novH	Medema et al. (2011)
Aminocoumarins	SpcD/SpcK-like thymidylyltransferase, aminocoumarins group	spcDK_like_cou	Medema et al. (2011)
Siderophores	Siderophore synthase	IucA_IucC	PFAM PF04183.5
Ectoines	Ectoine synthase	ectoine_synt	Medema et al. (2011)
Butyrolactones	AfsA-like butyrolactone synthase	AfsA	PFAM PF03756.6
Indoles	StaD-like chromopyrrolic acid synthase domain	indsynth	Medema et al. (2011)
Nucleosides	LipM-like nucleotidyltransferase	LipM	Medema et al. (2011)
Nucleosides	LipU-like protein	LipU	Medema et al. (2011)
Nucleosides	LipV-like dehydrogenase	LipV	Medema et al. (2011)
Nucleosides	ToyB-like synthase	ToyB	Medema et al. (2011)
Nucleosides	TunD-like putative N-acetylglucosamine transferase	TunD	Medema et al. (2011)
Nucleosides	Pur6-like synthetase	pur6	Medema et al. (2011)
Nucleosides	Pur10-like oxidoreductase	pur10	Medema et al.

Compound class	Description	HMM name	Source
			(2011)
Nucleosides	NikJ-like protein	nikJ	Medema et al. (2011)
Nucleosides	NikO-like enolpyruvyl transferase	nikO	Medema et al. (2011)
Phosphoglycolipids	MoeO5-like prenyl-3-phosphoglycerate synthase	MoeO5	Medema et al. (2011)
Phosphoglycolipids	Phosphoglycolipid glycosyltransferase	moeGT	Blin et al. (2013)
Melanins	MelC-like melanin synthase	melC	Medema et al. (2011)
Oligosaccharide	Secondary metabolite-related glycosyltransferase	Glycos_transf_1	PFAM PF00534.14
Oligosaccharide	Secondary metabolite-related glycosyltransferase	Glycos_transf_2	PFAM PF00535.20
Oligosaccharide	Secondary metabolite-related glycosyltransferase	Glyco_transf_28	PFAM PF03033.14
Oligosaccharide	Secondary metabolite-related glycosyltransferase	DUF1205	PFAM DUF1205
Oligosaccharide	Secondary metabolite-related glycosyltransferase	MGT	Blin et al. (2013)
Oligosaccharide	Secondary metabolite-related glycosyltransferase	MGT2	Blin et al. (2013)
Furan	MmyO-like protei	mmyO	Blin et al. (2013)
Homoserine lactone	Autoinducer synthetase	Autoind_synth	PFAM PF00765.12
Thiopeptide	YcaO-like	YcaO	PFAM PF02624.11
Phenazine	Phenazine biosynthesis gene	phzB	Blin et al. (2013)
Phosphonate	Phosphonate biosynthesis	phosphonates	Blin et al. (2013)
Others	NAD-binding domain 4	NAD_binding_4	PFAM PF07993.5
Others	LmbU-like protein	LmbU	Medema et al. (2011)
LAP	Goadsporin-like protein	goadsporin_like	Medema et al. (2011)
Others	Neocarzinostatin-like protein	Neocarzinostat	Medema et al. (2011)
Cyanobactin	Cyanobactin protease	cyanobactin_synth	Medema et al. (2011)
Others	Cyclodipeptide synthase	cycdipepsynth	Medema et al. (2011)
Others	Fom1-like phosphomutase	fom1	Medema et al. (2011)
Others	BcpB-like phosphomutase	bcpB	Medema et al. (2011)
Others	FrbD-like phosphomutase	frbD	Medema et al. (2011)
Others	MitE-like CoA-ligase	mitE	Medema et al. (2011)

Compound class	Description	HMM name	Source
Others	Valanimycin biosynthesis VlmB domain	vlmB	Medema et al. (2011)
Others	Pyrrolnitrin biosynthesis PrnB domain	prnB	Medema et al. (2011)
Others	Nitrosynthase domain	CaiA	Blin et al. (2013)
Others	Bacilysin-related ligase	bacilysin	Blin et al. (2013)

Within antiSMASH, the HMM profiles in Table S1 are used for detecting secondary metabolite biosynthetic gene clusters in genomic data.

Table S2: Rules for detection of secondary metabolite biosynthesis gene clusters (extended according to Medema et al., 2011, Blin et al., 2013)

Biosynthetic class	Rules
Type I PKS	- KS & AT HMM scores > 50 within one protein<br>- KS score > bactTypeIFAS / fungTypeIFAS / HglD&E / FabH scores
Trans-AT type I PKS	- trans-AT docking domain HMM score > 65<br>- KS score > 50<br>- no match to rules for normal type I PKSs as above
Type II PKS	- type II KS or CLF HMM score > 50<br>- KS/CLF score > enedyineKS / modularKS / hybridKS / iterativeKS / transATKS / bactTypeIFAS / fungTypeIFAS / TypeIIFAS / HglD&E / FabH HMM scores<br>- no match to rules for normal/trans type I PKSs as above
Type III PKS	- Chal_sti_synt_C or Chal_sti_synt_N HMM scores > 35<br>- no match to rules for normal type I&II PKSs as above
Type IV PKS	- HglE or HglD HMM scores > 50<br>- HglD/E HMM score > bactTypeIFAS / fungTypeIFAS / TypeIIFAS / FabH HMM scores<br>- no match to rules for normal type I&II&III PKSs as above
Non-ribosomal peptide synthetase	- C & A / A-OX domain HMM scores > 20 within one protein *OR* single domain C & A proteins scores > 20 within 20 kb distance
Terpene	- Terpene_Synth HMM score > 23 *OR* Terpene_Synth_C HMM score > 23 *OR* phytoene_synt HMM score > 20 *OR* Lycopene_cycl HMM score > 80 *OR* terpene_cyclase HMM score > 50 *OR* NapT7 HMM score > 250 *OR* fung_ggpps HMM score > 420 *OR* fung_ggpps2 HMM score > 312 *OR* dmat HMM score > 200 *OR* trichodiene_synth HMM score > 150 within 20 kb distance
Lanthipeptides	- (LANC_like HMM score > 80 and Lant_dehyd_N HMM score > 20 in one cluster) *OR* (LANC_like HMM score > 80 and Lant_dehyd_C HMM score > 20 in one cluster) *OR* (Lant_dehyd_N and Lant_dehyd_C HMM scores > 20 within one protein) *OR* (LANC_like HMM score > 80 and Flavoprotein HMM score > 20 in one cluster) *OR* (LANC_like HMM score > 80 and Trp_halogenase HMM score > 20 in one cluster) *OR* (LANC_like HMM score > 80 and p450 HMM score > 60 in one cluster) *OR* (LANC_like HMM score > 80 and adh_short HMM score > 100) *OR* (LANC_like HMM score > 80 and adh_short_C2 HMM score > 100) *OR* (LANC_like HMM score > 80 and Pkinase HMM score > 30 within one protein) OR (LANC_like HMM score > 80 and DUF4135 HMM score > 150 within one protein) OR one of a range of lantibiotic prepeptide HMM scores > 20 *OR* TIGR03731 HMM score > 18 within 20 kb distance
Bacteriocins	- Strepbact HMM score > 50 *OR* Antimicrobial14 HMM score > 90 *OR* Bacteriocin_IId HMM score > 23 *OR* BacteriocIIc_cy HMM score > 92 *OR* Bacteriocin_II HMM score > 40 *OR* Lactococcin HMM score > 24 *OR* Antimicrobial17 HMM score > 31 *OR* Lactococcin_972 HMM score > 25 *OR* Bacteriocin_IIc HMM score > 27 *OR* LcnG-beta HMM score > 78 *OR* Bacteriocin_IIi HMM score > 56 *OR* Cloacin HMM score > 27 *OR* Linocin_M18 HMM score > 25 *OR* TIGR03603 HMM score > 150 *OR* TIGR03604 HMM score > 440 *OR* TIGR03605 HMM score > 200 *OR* TIGR03651 HMM score > 18 *OR* TIGR03678 HMM score > 35 *OR* TIGR03693 HMM score > 400 *OR* TIGR03798 HMM score > 16 *OR* TIGR03882 HMM score > 150 *OR* TIGR03601 HMM score > 50 *OR*

Biosynthetic class	Rules
	TIGR03602 HMM score > 50 *OR* TIGR03795 HMM score > 41 *OR* TIGR03793 HMM score > 51 *OR* TIGR03975 HMM score > 282 *OR* mvnA HMM score > 20 *OR* DUF692 HMM score > 40 *OR*
Thiopeptide	- (Lant_dehyd_N HMM score > 20 *OR* Lant_dehyd_C HMM score > 20) and YcaO HMM score > 25 *OR* thiostrepton HMM score > 20 within 20 kb distance
Linaridin	- cypermycin HMM score > 10 *OR* cypI HMM score > 15
Cyanobactin	- cyanobactin_synth HMM score > 80
Glycocin	- glycocin HMM score > 30 *OR* sublancin HMM score > 30
LAP	- goadsporin_like HMM score > 500
Lasso peptide	- lasso HMM score > 400
Sactipeptide	- subtilosin HMM score > 140 *OR* thuricin HMM score > 30
Bottromycin	- botH HMM score > 65
Head-to-tail cyclised peptide	- Subtilosin_A HMM score > 98 *OR* skfc HMM score > 70
Microcin	- micJ25 HMM score > 21 *OR* mcjC HMM score > 60
Microviridin	- mvd HMM score > 20
Proteusin	- PoyD HMM score > 25
Beta-lactams	- Beta-lactam synthase HMM score > 250 *OR* clavulanic acid synthase HMM score > 250 *OR* tabtoxin synthase score > 500
Aminoglycosides / aminocyclitols	- strH HMM score > 50 *OR* strK1 HMM score > 800 *OR* strK2 HMM score > 650 *OR* NeoL HMM score > 50 *OR* DOIS HMM score > 500 *OR* ValA HMM score > 600 *OR* SpcFG HMM score > 200 *OR* SpcDK_glyc HMM score > 600 *OR* salQ HMM score > 480
Aminocoumarins	- novK HMM score > 200 *OR* novJ HMM score > 350 *OR* novI HMM score > 600 *OR* novH HMM score > 750 *OR* spcDK_like_cou HMM score > 600
Siderophores	- IucA_IucC HMM score > 30
Ectoines	- Ectoine_synt HMM score > 35
Butyrolactones	- AfsA HMM score > 25
Indoles	- ind_synth HMM score > 100
Nucleosides	- LipM HMM score > 50 *OR* LipU HMM score > 30 *OR* LipV HMM score > 375 *OR* ToyB HMM score > 175 *OR* TunD HMM score > 200 *OR* pur6 HMM score > 200 *OR* pur10 HMM score > 600 *OR* nikJ HMM score > 200 *OR* nikO HMM score > 400
Phosphoglycolipids	- MoeO5 HMM score > 65 *OR* moeGT HMM score > 40
Melanins	- melC HMM score > 40
Oligosaccharide	- at least three out of (Glycos_transf_1 HMM score > 20, Glycos_transf_2 HMM score > 20, Glyco_transf_28 HMM score > 26, MGT HMM score > 100, MGT2 HMM score > 150, DUF1205 HMM score > 20)
Furan	- mmyO HMM score > 500
Homoserine lactone	- Autoind_synth HMM score > 20
Phenazine	- phzB HMM score > 80
Phosphonate	- phosphonates HMM score > 275
Others	- PP-binding & AMP-binding HMM scores > 20 within one protein *OR* (PP-binding HMM score > 20 and A-OX HMM score > 50 within one protein) *OR* (NAD_binding_4 HMM score > 40 and A-OX HMM score > 50 within one protein) *OR* (NAD_binding_4 HMM score > 40 and AMP-binding HMM score > 20 within one protein) *OR* LmbU HMM score > 50 *OR* Neocarzinostat HMM score > 28 *OR* *OR* cycdipepsynth HMM score > 110

Biosynthetic class	Rules
	OR fom1 HMM score > 750 *OR* bcpB HMM score > 400 *OR* frbD HMM score > 350 *OR* mitE HMM score > 400 *OR* vlmB HMM score > 250 *OR* prnB HMM score > 200 or CaiA HMM score > 200 or bacilysin HMM score > 160
ClusterFinder Saccharide	- At least two out of (Glycos_transf_1 HMM score > 20 *OR* Glycos_transf_2 HMM score > 20 *OR* Glyco_transf_28 HMM score > 26 *OR* PF02922 HMM score > 20 *OR* PF01041 HMM score > 19 *OR* PF00128 HMM score > 20 *OR* PF00908 HMM score > 20 *OR* PF02719 HMM score > 20 *OR* PF04321 HMM score > 20 *OR* PF01943 HMM score > 203*OR* PF02806 HMM score > 20 *O1* PF02350 HMM score > 22 *OR* PF02397 HMM score > 21 *OR* PF04932 HMM score > 24 *OR* PF01075 HMM score > 20 *OR* PF00953 HMM score > 21 *OR* PF01050 HMM score > 23 *OR* PF01501 HMM score > 24 *OR* PF05159 HMM score > 20 *OR* PF04101 HMM score > 21 *OR* PF02563 HMM score > 26 *OR* PF08437 HMM score > 19 *OR* PF02585 HMM score > 22) within 20 kb of each other
ClusterFinder fatty acid	- bt1fas HMM score > 50 *OR* ft1fas HMM score > 69 *OR* t2fas or fabH HMM score > 50

Table S3: HMM library for PKS/NRPS domain architecture (extended according to Medema et al., 2011)

Compound class	Description	HMM name	Source
NRPS	Condensation domain	Condensation	PFAM PF00668.13
NRPS	Condensation domain that links L-amino acid to peptide ending with D-amino acid	Condensation_DCL	Rausch et al. (2007)
NRPS	Condensation domain that links L-amino acid to peptide ending with L-amino acid	Condensation_LCL	Rausch et al. (2007)
NRPS	Dual condensation / epimerization domain	Condensation_Dual	Rausch et al. (2007)
NRPS	Starter condensation domain	Condensation_Starter	Rausch et al. (2007)
NRPS	Putatively inactive glycopeptide condensation-like domain	X (formerly CXglyc)	Medema et al. (2011)
NRPS	Glycopeptide condensation domain	Cglyc	Medema et al. (2011)
NRPS	Heterocyclization domain	Heterocyclization	Medema et al. (2011)
NRPS	Epimerization domain	Epimerization	Weber et al. (2009)
NRPS	Adenylation domain	AMP-binding	PFAM PF00501.21
NRPS	Adenylation domain with integrated oxidase	A-OX	Medema et al. (2011)
NRPS	Peptidyl-carrier protein domain	PCP	Medema et al. (2011)
NRPS	NRPS COM domain N-terminal	NRPS-COM_Nterm	Medema et al. (2011)
NRPS	NRPS COM domain C-terminal	NRPS-COM_Cterm	Medema et al. (2011)
NRPS	4'-phosphopantetheinyl transferase	ACPS	PFAM PF01648.9
PKS	Ketosynthase domain	PKS_KS	SMART
PKS	Acyltransferase domain	PKS_AT	SMART
PKS	Ketoreductase domain	PKS_KR	SMART
PKS	Enoylreductase domain	PKS_ER	SMART
PKS	Dehydrogenase domain	PKS_DH	SMART
PKS	Acyl-carrier protein domain	ACP	Medema et al. (2011)
PKS	Beta-branching acyl-carrier protein domain	ACP_beta	SMART
PKS	Trans-acyltransferase docking domain	Atd	Medema et al. (2011)
PKS	Enediyine ketosynthase	Ene_KS	Yadav et al. (2009)
PKS	Modular ketosynthase	Mod_KS	Yadav et al. (2009)

PKS	Hybrid ketosynthase	Hyb_KS	Yadav et al. (2009)
PKS	Iterative ketosynthase	Itr_KS	Yadav et al. (2009)
PKS	Trans-AT ketosynthase	Tra_KS	Yadav et al. (2009)
PKS	Polyketide cyclase	Polyketide_cyc	PFAM PF03364.13
PKS	Polyketide cyclase / dehydratase	Polyketide_cyc2	PFAM PF10604.2
PKS	PKS N-terminal docking domain	PKS_Docking_Nterm	Medema et al. (2011)
PKS	PKS C-terminal docking domain	PKS_Docking_Cterm	Medema et al. (2011)
PKS	Co-enzyme A ligase domain	CAL	Medema et al. (2011)
PKS	Branching domain	B	This study.
PKS	FkbH-like domain	FkbH	This study.
PKS	GNAT domain	GNAT	This study.
PKS	Halogenase domain	Hal	This study.
PKS	Pyran synthase domain	PS	This study.
PKS	Crotonase / Enoyl-CoA hydratase domain	CR	This study.
NRPS/PKS	Formylation domain	F	This study.
NRPS/PKS	C-methyl transferase	cMT	Ansari et al. (2008)
NRPS/PKS	O-methyl transferase	oMT	Ansari et al. (2008)
NRPS/PKS	N-methyl transferase	nMT	Ansari et al. (2008)
NRPS/PKS	Aminotransferase class I&II	Aminotran_1_2	PFAM PF00155.14
NRPS/PKS	Aminotransferase class III	Aminotran_3	PFAM PF00202.14
NRPS/PKS	Aminotransferase class IV	Aminotran_4	PFAM PF01063.12
NRPS/PKS	Aminotransferase class V	Aminotran_5	PFAM PF00266.12
NRPS/PKS	Thiolation domain	PP-binding	PFAM PF00550.18
NRPS/PKS	Thioesterase domain	Thioesterase	PFAM PF00975.13
NRPS/PKS	Terminal reductase domain	TD	Medema et al. (2011)

The HMM profiles in Table S3 are used to detect the different domains of NRPS and PKS for detailed annotation.

Supplementary references

1. Medema, M.H., Blin, K., Cimermancic, P., de Jager, V., Zakrzewski, P., Fischbach, M.A., Weber, T., Takano, E., and Breitling, R. (2011) antiSMASH: rapid identification, annotation and analysis of secondary metabolite biosynthesis gene clusters in bacterial and fungal genome sequences. *Nucl. Acids Research* **39**, W339-W346.
2. Blin, K., Medema, M.H., Kazempour, D., Fischbach, M.A., Breitling, R., Takano, E., and Weber, T. (2013) antiSMASH 2.0 – a versatile platform for genome mining of secondary metabolite producers. *Nucl. Acids Research* **41**, W204-212.
3. Yadav, G., Gokhale, R.S. and Mohanty, D. (2009) Towards prediction of metabolic products of polyketide synthases: an in silico analysis. *PLoS Comput. Biol.*, **5**, e1000351.
4. de Jong, A., van Heel, A.J., Kok, J. and Kuipers, O.P. (2010) BAGEL2: mining for bacteriocins in genomic data. *Nucleic Acids Res.*, **38**, W647-651.
5. Rausch, C., Hoof, I., Weber, T., Wohlleben, W. and Huson, D.H. (2007) Phylogenetic analysis of condensation domains in NRPS sheds light on their functional evolution. *BMC Evol. Biol.*, **7**, 78.
6. Ansari, M.Z., Sharma, J., Gokhale, R.S. and Mohanty, D. (2008) In silico analysis of methyltransferase domains involved in biosynthesis of secondary metabolites. *BMC Bioinformatics*, **9**, 454.
